# The influence of the pH on the incorporation of caffeic acid into biomimetic membranes and cancer cells

**DOI:** 10.1038/s41598-022-07700-8

**Published:** 2022-03-07

**Authors:** Monika Naumowicz, Magdalena Kusaczuk, Marcin Zając, Agata Jabłońska-Trypuć, Agnieszka Mikłosz, Miroslav Gál, Mateusz Worobiczuk, Joanna Kotyńska

**Affiliations:** 1 Department of Physical Chemistry, Faculty of Chemistry, University of Bialystok, Ciolkowskiego 1K, 15-245 Białystok, Poland; 2 Department of Pharmaceutical Biochemistry, Medical University of Bialystok, Mickiewicza 2A, 15-222 Białystok, Poland; 3 Doctoral School of Exact and Natural Sciences, University of Bialystok, Ciolkowskiego 1K, 15-245 Białystok, Poland; 4 Department of Chemistry, Biology and Biotechnology, Bialystok University of Technology, Wiejska 45A, 15-351 Białystok, Poland; 5 Department of Physiology, Medical University of Bialystok, Mickiewicza 2C, 15-222 Białystok, Poland; 6 Department of Inorganic Technology, Faculty of Chemical and Food Technology, Slovak University of Technology, Radlinského 9, 812 37 Bratislava, Slovakia

**Keywords:** Biophysics, Chemical biology

## Abstract

Caffeic acid (CA) is a phenolic compound synthesized by all plant species. It constitutes the main hydroxycinnamic acid found in human diet and presents a variety of beneficial effects including anticancer activity. Current data suggests essential role of the interplay between anticancer drugs and the cell membrane. Given this, biophysical interactions between CA and cancer cells or biomimetic membranes were investigated. Glioblastoma cell line U118MG and colorectal adenocarcinoma cell line DLD-1, as well as lipid bilayers and liposomes, were used as in vitro models. Electrophoretic light scattering was used to assess the effect of CA on the surface charge of cancer cells and liposomal membranes. Electrochemical impedance spectroscopy was chosen to evaluate CA-dependent modulatory effect on the electrical capacitance and electrical resistance of the bilayers. Our results suggest that CA fulfills physicochemical criteria determining drug-like properties of chemical compounds, and may serve as a potential cytostatic agent in cancer treatment.

## Introduction

Caffeic acid (CA) ((E)-3-(3,4-dihydroxyphenyl)prop-2-enoic acid, 3,4-dihydroxycinnamic acid) is a naturally occurring hydroxycinnamic acid synthesized by all plant species. CA constitutes the main hydroxycinnamic acid found in the diet of humans^1,2^. Many staple foods such as apples, blueberries, sweet potatoes, carrots, mushrooms, tea, coffee drinks, cider, wine, olive oil, and numerous culinary herbs are among the richest sources of CA^2–6^.

One of the best documented biological properties of CA is the antioxidant activity. It is specifically important for the food industry as it makes this polyphenol an efficient compound preventing oxidation processes and prolonging food stability. Additionally, other biological effects with impact on human health have been reported for CA. These include anti-inflammatory, anti-atherosclerotic, immunomodulatory and antimutagenic activities, as well as chemoprevention and pro-apoptotic effect in cancer cells^2,4,5,7^. Due to the high chemical versatility and modifiability of CA, it is now considered a promising compound with favoring chemical structure in drug discovery programs^7^.

The plasma membrane is a complex and dynamic structure composed of multiple lipids and membrane proteins. The lipid profile of the plasma membrane is asymmetric and considered to be cell type-specific^8^. A loss of this asymmetry has been reported for lipid bilayers of several cancer cell types, resulting in the exposition of the anionic phosphatidylserine PS on the surface of their membranes^9^. This PS excess may thus be regarded as a tumor marker^10^. Such reorganization of lipid bilayer may alter certain properties of biomembranes such as their fluidity and plasticity, or perturb cell signaling^11,12^. Moreover, altered phospholipid distribution may affect electrochemical parameters of cell membrane resulting in disrupted cell functioning and impaired sensitivity to anticancer drugs^13–16^.

Importantly, modification of the physicochemical properties of cancer cells applies not only to the cell membrane itself but also occurs in their surrounding environment^17^. It has been demonstrated that acidic extracellular pH is a hallmark of the tumor phenotype and is beneficial for malignant cells as it promotes invasiveness^17^. This is particularly significant because it may affect the ionization state of drugs, and therefore be a key factor in their partitioning into the membrane. The amount of ionized and non-ionized forms of a drug depends on its ionization constant and the pH of the medium. Therefore, anticancer drugs can interact with cell membranes differently depending on their charge state, which is strictly related to the pH of their microenvironment. In this respect, analysis of various physicochemical parameters of living cells has become a strong trend in modern oncology and oncopharmacology^9^. It has been already well established, that lipids can be used as targets to overcome drug resistance in cancer^18^. Moreover, membranes can also determine drug penetration, conformation, and/or localization in the membrane, and subsequently, affect their therapeutic targets. Conversely, drugs also exert various effects at the membrane level, which include alterations in lipid conformation, surface charge density (*δ*), lipid domains, membrane fluidity, and eventually cell functioning^19^. Therefore, characterization and understanding of the interactions between chemotherapeutic compounds and cell membranes is an important biophysical problem.

As a result of the complexity of cell membranes, numerous mimetic model systems and biophysical methods have been established and applied to investigate specific interactions between anticancer drugs and membrane lipids. The implementation of biomimetic membranes allows to create membranes of desired structure and complexity by altering the lipid composition. Simplification of membrane systems, and hence full control of these structures is critical for understanding the interactions at the molecular level. The most basic model of a natural membrane that is often used for experimental applications is the bilayer lipid membrane (also called black lipid bilayer or just BLM). BLM formation methods assure easy access to both sides of the bilayer reflecting the inside of the cell and its surroundings. Another biomimetic system often used in cell research is based on liposomes. Amongst liposomes, the small unilamellar vesicles (SUVs) and large unilamellar vesicles (LUVs) share similar curvature with the cell membrane, which makes them commonly used type of liposomes in cell-based studies. Since liposomes are biocompatible, fully biodegradable, non-immunogenic and non-toxic systems, they are suitable for the delivery of hydrophobic, amphipathic and hydrophilic drugs.

Recently, the main line of investigation of our team has been focused on the analysis of natural polyphenols on electrical properties of BLMs, liposomes and cancer cells in vitro. Given this, we already managed to identify significant changes in electrical capacitance, electrical resistance, and surface charge of model and natural membranes upon their structural modification by quercetin^20^ as well as cinnamic, ferulic, and p-coumaric acids^13–16^. Herein, we evaluated the cytotoxic potential of CA against human glioblastoma cell line U118MG and human colorectal adenocarcinoma cell line DLD-1 and described physicochemical changes occurring in membranes of living cells. In addition to the analyses on the cellular level, we evaluated the modulatory effect of CA on the electrical capacitance and electrical resistance of BLMs and the surface charge density of liposomes. To describe these parameters the electrochemical impedance spectroscopy (EIS) and electrophoretic light scattering (ELS) techniques have been used. We demonstrated that modification of the pH of the environment surrounding the membrane results in differential electrical charge values accumulated on the membrane surface suggesting the highest intracellular intake and lowest surface adsorption of CA in pH below p*K*_COOH_ value. Therefore, the most efficient intramembranous delivery of CA seems to coincide with low pH values of the solution. These results encourage further investigation of potential solvents/co-therapeutics lowering the pH for better CA intracellular permeability and higher therapeutic efficiency. The combination of cell-based and electrochemical methods may bring further insight into the complicated nature of interactions between therapeutic agents and membranes—natural barriers for intracellular penetration of drugs. Determination of CA-dependent cytotoxic effect followed by identification of changes in physicochemical parameters of membranes may be a good predictor of the in vivo bioavailability of anticancer agents and might serve as a promising tool in drug design in pharmaceutical sciences.

## Materials and methods

### Reagents and chemicals

Powdered 1,2-dioleoyl-sn-glycero-3-phosphocholine (purity > 99%) and 1,2-diacyl-sn-glycero-3-phospho-l-serine (purity ≥ 97%) were purchased from Sigma-Aldrich (St. Louis, MO, USA) and stored at − 20 °C. The caffeic acid (purity ≥ 98.0%) and methylthiazolyldiphenyl-tetrazolium bromide (MTT) were also provided by Sigma-Aldrich (St. Louis, MO, USA). The Dulbecco's modified Eagle's medium (DMEM) was obtained from Gibco (San Diego, CA, USA).

The electrolyte solutions (155 mmol/L NaCl) and cleaning procedures were done using ultrapure water (resistivity of 18.2 MΩ) from HLP 5UV System (Hydrolab, Hach Company, Loveland, CO, USA).

### Methods

#### Cell cultures

Human glioblastoma cell line U118MG, and human colorectal adenocarcinoma cell line DLD-1 were provided by American Type Culture Collection (ATCC). Cells were maintained in high glucose DMEM with addition of 10% of heat-inactivated fetal bovine serum Gold (FBS Gold), penicillin (100 U/mL), streptomycin (100 μg/mL), and 2 mmol/mL L-glutamine. The cells were cultured in Falcon flasks (BD Pharmingen™, San Diego, CA, USA) in Galaxy 170R incubator (Eppendorf Inc., Hamburg, Germany) in a humidified atmosphere of 5% CO_2_ in the air, at 37 °C. Cells reaching sub-confluency were detached from the culture dishes using 0.05% trypsin, 0.02% EDTA in calcium-free phosphate-buffered saline (PBS) and counted in Luna-II™ automated cell counter (Logos Biosystems, Annandale, VA, USA). CA was dissolved in dimethyl sulfoxide (DMSO) and subsequently diluted in growth medium keeping the final concentration of DMSO ≤ 0.5% in cultures.

#### Cell viability

The cells viability was measured according to the method described in our previous works^15,20,21^ using 3-(4,5-dimethylthiazol-2-yl)-2,5-diphenyltetrazoliumbromide (MTT). In brief, cells were seeded in 96-well plates at a density of 2 × 10^4^ cells/well in 200 µL of culture medium. Confluent cells were exposed to various concentrations of CA (0.2–7 mmol/L) for 24 and 48 h. After an intended time of incubation, cells were washed 3 times with PBS and incubated with 100 µL of MTT solution (0.5 mg/mL in PBS) at 37 °C in a humidified 5% CO_2_ atmosphere for 2 h. The medium was removed and formazan products were solubilized in 100 µL of DMSO and incubated in the dark for an additional 2 h. The absorbance of a converted dye in living cells was read at λ = 570 nm using a microplate reader GloMax®-Multi Microplate Multimode Reader (Promega Corporation, Madison, WI, USA). The viability of CA-treated cells was calculated as a percentage of control cells incubated without tested compound.

#### Preparation of liposomes

Liposomes were prepared by the thin-film hydration method followed by extrusion to reduce their size and obtain SUVs. Briefly, phospholipids: DOPC, PS or 3DOPC/1PS (w/w) were dissolved in chloroform. The solvent was completely evaporated under argon gas, which assured formation of a thin film. Resultant lipid films were hydrated by the addition of an electrolyte solution (155 mmol/L NaCl), and shaken for 20 min to create multilamellar vesicles. The process was conducted in temperatures exceeding those known as the phase transition temperatures of the solid lipids. As such, the temperature of − 16.5 °C was chosen for DOPC^22,23^ and the temperature of 68 °C was selected for PS extract. The PS-specific temperature was adopted from the previously reported data, and the possible presence of phosphatidylserines with different phase transition temperatures (from − 11 °C for DOPS to 68 °C for DSPS^23,24^) in the extract was taken into account. Then, the multilamellar vesicle solution was subjected to ultrasonication to receive SUVs. In this respect, the large unilamellar vesicles solution (100 μg/mL) was passed through a polycarbonate membrane of 100 nm porosity ((Millipore, Darmstadt, Germany) at least eleven times and mounted in a mini-extruder (Avanti Polar Lipids, Inc., Alabaster, AL) fitted with two 1000-μL Hamilton gas tight syringes. The addition of CA to liposomes (1 mmol/L) was preceded by dissolving the compound in the chloroform. Next, chloroform-dissolved CA was added to the phospholipids before the preparation of the thin lipid film. For Dynamic Light Scattering (DLS) measurements, an aliquot of CA was added to liposome suspensions after extrusion.

#### Preparation of bilayer lipid membranes

DOPC, PS and CA were weighed, dissolved in chloroform, and mixed in appropriate amounts. Chloroform was removed under a gentle stream of argon to obtain a dry film. Next, the film was dissolved in a n-hexadecane-n-butanol mixture (10:1 by volume). The final concentration of lipids was 20 mg/mL in solvent system. The BLM-forming solutions contained DOPC, 3DOPC/1PS (w/w) or DOPC-CA and 3DOPC/1PS-CA mixtures (1 mmol/L concentration of phenolic acid with respect to the phospholipid). All the solutions were stored in the dark under nitrogen at a temperature of 4 °C for a minimum of 3 days before investigations.

BLMs were created according to the method reported in our previous study^25^. They were obtained on a Teflon plug constituting a component of the measuring vessel. Bilayer formation process was controlled electrically by measuring the membrane capacitance at low frequency and optically by reflected light microscopy with a high-brightness yellow LED source. Photographs of the membranes were captured with a color CCD camera with the use of WinFast PVR program. Membrane areas were determined from the photographs, accounting the spherical nature of the surfaces and utilizing the Macroaufmassprogram^26^. The area of the bilayers was approximately 6 × 10^−2^ cm^2^.

#### Electrophoretic light scattering measurements

The zeta potential (*ζ*) and the surface charge density of the liposomes and cell membranes were obtained by performing microelectrophoretic assessments of the samples using ELS technique. The measurements were conducted by a Malvern Zetasizer Nano ZS system (Malvern Instruments, Malvern, United Kingdom) in folded capillary cells (Malvern DTS 1070).

For cell-based analysis U118MG and DLD-1 cells (1 × 10^5^ cells/well) were cultured in 6-well plates and incubated in 2 mL of culture medium with 1 mmol/L and 4 mmol/L CA respectively, for 24 and 48 h. Control cells were seeded and cultured without the tested compound. After an intended time of incubation medium was aspirated, cells were washed with 155 mmol/L NaCl, scraped with a cell scraper, and suspended in 2 mL of cold 155 mmol/L NaCl. Next, cells were subjected to ELS analysis.

Both liposomes and cells suspended in 155 mmol/L NaCl solution were titrated to the desired pH (range 2–10, every ± 0.3 units) using HCl or NaOH. Six electrophoretic mobility measurements were made (each covering 100–200 runs with duration of 2 s) for each pH value per sample. Each sample was measured in triplicate and the mean values are presented.

The experimental *δ* was determined as previously described^20^, from the electrophoretic mobility using the following equation:

1 $$ \delta = \frac{\eta \cdot u}{d} $$

where *η*—the viscosity of the solution, *u*—the electrophoretic mobility, *d*—the diffuse layer thickness.

The *d* was calculated according to the equation presented below^27^:

2 $$ d = \sqrt {\frac{{\varepsilon \cdot \varepsilon_{0} \cdot R \cdot T}}{{2 \cdot F^{2} \cdot I}}} $$

where *R*—the gas constant, *T*—the temperature, *F*—the Faraday constant, *I*—the ionic strength of 155 mmol/L NaCl, and *ε* and *ε*_0_ refer to the permittivity of free space and the relative permittivity of the medium.

The electrophoretic mobility was converted to the *ζ* using Henry’s equation^28^:

3 $$ \zeta = \frac{3\mu \eta }{{2_{{\varepsilon \varepsilon_{0} }} f\left( {ka} \right)}} $$

where *a*—the particle radius, *κ*^−1^—the Debye length, ƒ(κa)—Henry’s function.

The mathematical model which allows to determine the theoretical *δ* of natural cell membranes was reported in detail in previous studies^14,29^. This model assumes the existence of four membrane surface equilibria with H^+^, OH^−^, Na^+^ and Cl^−^ ions, and the final equation characterizing the *δ* can be expressed as follows:

4 $$ \frac{\delta }{F} = \frac{{C_{TB} \cdot a_{{H^{ + } }} }}{{\left( {1 + K_{BCl} \cdot a_{{cl^{ - } }} } \right) + K_{BOH} \cdot K_{W} }} - \frac{{C_{TA} }}{{K_{AH} \cdot a_{{H^{ + } }} + K_{ANa} \cdot a_{{Na^{ + } }} + 1}} $$

where *C*_TA_—total surface concentration of the membrane acidic groups; *C*_TB_—total surface concentration of the membrane basic groups; *K*_AH_, *K*_ANa_, *K*_BOH_, *K*_BCl_—associations constants; *F*—the Faraday constant.

Equation (4) must be simplified to a linear form at low H^+^ (*a*_H_^+^  → 0) and high H^+^ (*a*_H_^+^  → ∞) concentrations to determine the appropriate parameters. The simplification of the equation results in two linear dependencies, one correct for low H^+^ ion concentration:

5 $$ \frac{\delta }{F}a_{{{\text{H}}^{ + } }}^{ - 1} = \frac{{ - C_{{{\text{TA}}}} \cdot a_{{{\text{H}}^{ + } }}^{ - 1} }}{{1 + K_{{{\text{ANa}}}} \cdot a_{{{\text{Na}}^{ + } }} }} + \left( {\frac{{C_{{{\text{TB}}}} }}{{K_{{{\text{BOH}}}} \cdot K_{{\text{W}}} }} + \frac{{K_{{{\text{AH}}}} \cdot C_{{{\text{TA}}}} }}{{(1 + K_{{{\text{ANa}}}} \cdot a_{{{\text{Na}}^{ + } }} )^{2} }}} \right) $$

and the other for high H^+^ ion concentration:

6 $$ \frac{\delta }{F}a_{{{\text{H}}^{ + } }} = \frac{{C_{{{\text{TB}}}} }}{{1 + K_{{{\text{BCl}}}} \cdot a_{{{\text{Cl}}^{ - } }} }} \cdot a_{{{\text{H}}^{ + } }} - \left( {\frac{{K_{{{\text{BOH}}}} \cdot K_{{\text{W}}} \cdot C_{{{\text{TB}}}} }}{{(1 + K_{{{\text{BCl}}}} \cdot a_{{{\text{Cl}}^{ - } }} )^{2} }} + \frac{{C_{{{\text{TA}}}} }}{{K_{{{\text{AH}}}} }}} \right) $$

From the relationships above, the intercepts and slopes can be easily determined graphically. The coefficients obtained from the linear regression can be used to calculate *K*_AH_, *K*_BOH_, *C*_TA_, and *C*_TB_ values. Determination of the values of these parameters enables subsequent calculation of the theoretical values of cell membrane surface charge from Eq. (4) in comparison to experimental data.

#### Dynamic light scattering measurements

The size and the polydispersity index (PDI) of the liposomes were measured using DLS technique on a Malvern Zetasizer Nano ZS system (Malvern Instruments, Malvern, United Kingdom) at 25 °C. Size measurements were performed in disposable folded capillary cells (Malvern DTS0012) using an angle of detection of 173° backscatter. All experiments were conducted three times on each sample to check for results repeatability. Obtained data were analyzed using Zetasizer software.

#### Electrochemical impedance spectroscopy measurements

For the EIS measurements, an Autolab potentiostat (model PGSTAT302N, Metrohm, Utrecht, the Netherlands) equipped with a FRA2 module was used. A four-probe cell with two identical reversible Ag/AgCl electrodes with a salt bridge and two identical platinum electrodes were used to carry the current for the measurements. The electrochemical cell was comprehensively described in our previous work^25^. The EIS measurements were carried out at open circuit potential in the frequency ranging from 0.1 to 100,000 Hz with a sinusoidal voltage excitation of 4 mV amplitude. The 155 mmol/L NaCl electrolyte solutions of pH 3.0 and 7.4 were used to register impedance spectra (the pH was adjusted to the final value by adding concentrated sodium hydroxide or hydrochloric acid solutions).

**Scheme 1:**
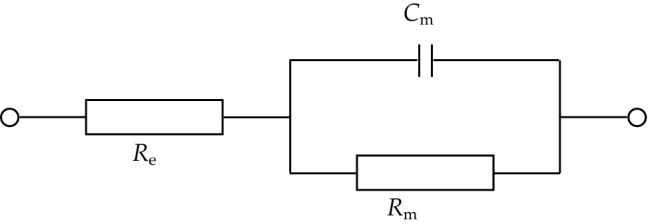
The equivalent circuit used to fit the data.

All impedance data were analyzed using ‘*Fit and simulation’* command in the NOVA software (v. 1.10). The equivalent circuit provided in Scheme 1 was used to fit the experimental data. In this circuit, *R*_e_ represents the uncompensated resistance in the circuit with regard to the solution resistance and the resistances associated with the leads and contacts; *C*_m_ is the membrane capacitance, and *R*_m_ is the membrane resistance. If the lipid bilayer is modified by compounds, e.g. channel formers or carriers, that increase its conductivity, the equivalent electrical circuit is more complex^30^. The values of the impedance parameters were normalized to the membrane area*,* representing the average of six independent measurements.

#### Statistical analysis

Cell-based data are expressed as mean ± SD from three independent experiments run in triplicate. GraphPad Prism 5.0 software (La Jolla, CA, USA) was used to perform statistical analyses. One-way analysis of variance (ANOVA) was carried out for comparisons between control and treated groups. Pair-wise comparisons were made by the post hoc Tukey’s test. Post hoc tests were run only if *F* achieved the necessary level of statistical significance and there was no significant variance inhomogeneity. Group differences were considered statistically significant for *p < 0.05. Electrochemical results are reported as means ± SD from six independent measurements for EIS and three independent measurements for ELS and DLS; obtained data were analyzed using standard statistical analyses, one way ANOVA with Scheffe's F test for multiple comparisons to determine significance between different groups. Statistical significance was defined as *p < 0.05, **p < 0.01.

## Results

In order to get a deeper insight into the influence of pH on the interactions between CA and biomimetic membranes or living cells, a systematic series of experiments was carried out. Firstly, the antiproliferative effect of CA on U118MG and DLD-1 cell lines was determined. Next, the half-maximal inhibitory concentration (IC_50_) values were calculated and all electrochemical measurements were performed in concentrations reflecting approximate IC_50_ values. The *δ* of both the intact cells and cells treated with CA was determined using the ELS technique. Subsequently, *δ* of pure and CA-modified phospholipid liposomes was determined for comparative purposes. Liposomes were prepared from DOPC, PS or a mixture of 3DOPC/1PS (w/w). Afterwards, the *C*_m_ and the *R*_m_ of DOPC or 3DOPC/1PS BLMs pure and modified with CA were determined based on the EIS spectra.

### Effect of caffeic acid on viability of human glioblastoma and human colorectal adenocarcinoma cell membranes

**Figure 1:**
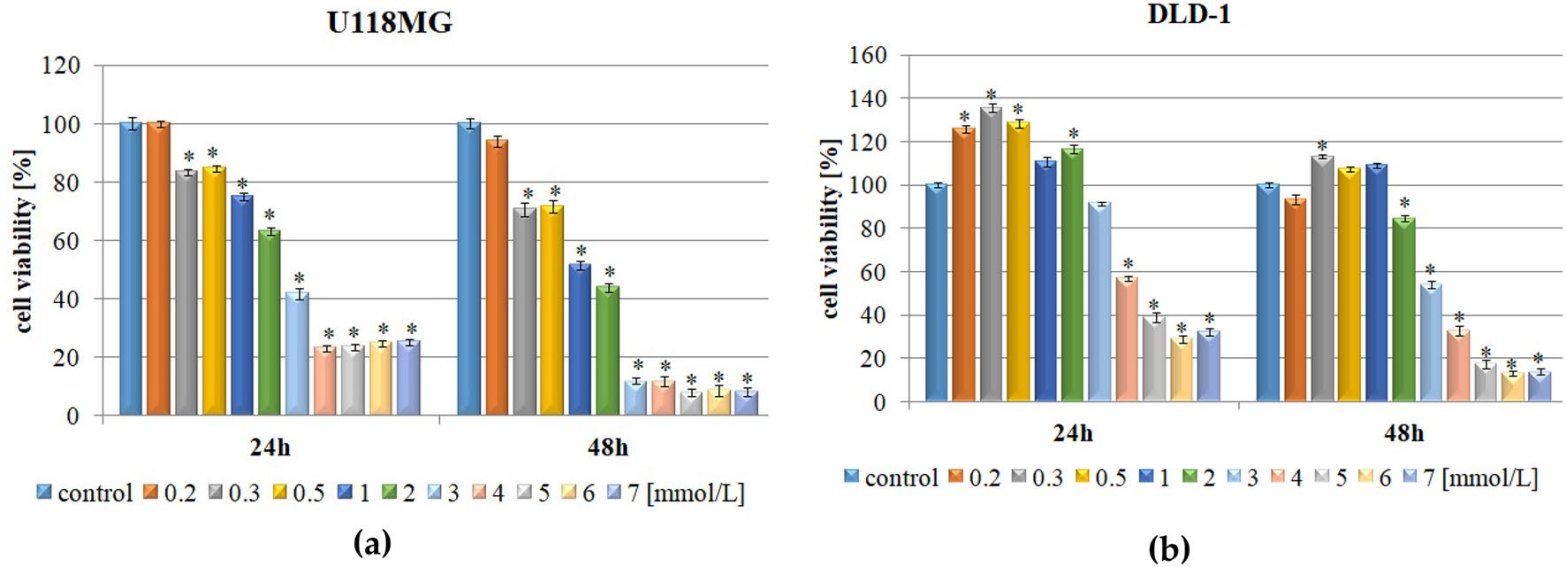
Cytotoxic effect of different concentrations of caffeic acid against U118MG (**a**) and DLD-1 (**b**) cells. Values are expressed as means ± SD from three individual experiments run in triplicates. *Indicates statistical significance (*p* ≤ 0.05) in comparison to untreated control cells (0 mmol/L).

Antiproliferative effects of CA on glioblastoma U118MG and colorectal adenocarcinoma DLD-1 cell lines were assessed using MTT assay. Cells were exposed to increasing concentrations of CA (0.2–7 mmol/L), for 24 h and 48 h. For both cell lines CA caused dose- and time-dependent reduction in cell viability (Fig. 1). However, low concentrations of CA had a relatively low ability to limit cell proliferation with the most efficient cytotoxic effect in U118MG (Fig. 1a), and hardly any antipoliferative activity or even growth-stimulatory effect in DLD-1 (Fig. 1b). The viability of glioblastoma cell line was inhibited as soon as 24 h after exposure to CA and after stimulation with the dose as low as 0.3 mmol/L (Fig. 1a). This effect was even more pronounced in cultures treated for 48 h, reaching 82% up to 92% of unviable cells in CA concentrations ranging from 3 to 7 mmol/L (Fig. 1a). In comparison, colorectal adenocarcinoma DLD-1 cells exposed to CA showed significant cytotoxic effects, approaching nearly 90% unviable cells, at the highest concentrations of CA (5–7 mmol/L) after 48 h of treatment. In this case, dose-dependent loss of viability was most apparent (Fig. 1b).

In order to compare the sensitivity of glioblastoma and colorectal carcinoma cells to CA, the GraphPad Prism 5 software was used to calculate the IC_50_ values after 48 h of treatment. The results showed significantly different IC_50_ values of approximately 0.944 mmol/L, and 3.208 mmol/L for U118MG and DLD-1 cells, respectively. Further cell-based measurements were carried out in concentrations slightly exceeding calculated IC_50_ values (1 mmol/L for U118MG and 4 mmol/L for DLD-1).

### Effect of caffeic acid on the membranes surface charge of human glioblastoma and human colorectal adenocarcinoma cells

**Figure 2:**
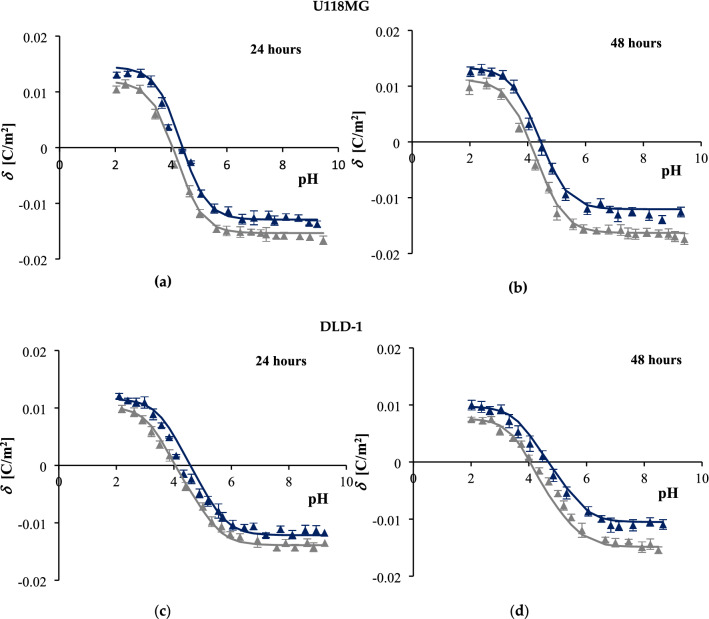
The membrane surface charge density of U118MG glioblastoma cells (**a**,**b**) and DLD-1 colon adenocarcinoma cells (**c**,**d**) at different pH values. Control cells are represented by grey and CA-treated cells by navy blue markings. Cells were treated for 24 h (**a**,**c**) and 48 h (**b**,**d**).

The surface charge density of cell membranes cannot be directly measured. However, this parameter can be extracted from electrophoretic mobility data. The most common technique that enables the measurement of the electrophoretic mobility of cells is ELS. To investigate the influence of CA on the electric properties of human glioblastoma U118MG and human colorectal adenocarcinoma DLD-1 cell lines, a series of microelectrophoretic experiments in a function of pH was performed. Based on the results of MTT analysis ("Effect of caffeic acid on viability of human glioblastoma and human colorectal adenocarcinoma cell membranes" Section), CA in a concentration of 1 mmol/L was chosen to treat glioblastoma cells, and a concentration of 4 mmol/L was chosen to treat colon cancer cells. Both U118MG and DLD-1 cells were incubated with CA for 24 and 48 h. The experimental *δ* data were calculated from Eq. (1). Theoretical values of membrane *δ* for both analyzed cell lines were calculated using Eq. (4) and further compared with experimental ones by putting them onto one graph (Fig. 2).

Figure 2 presents the influence of CA on the surface charge density of U118MG and DLD-1 cell lines respectively. The experimental data are presented as points, whereas the theoretical ones are marked with lines. Notably, obtained curves present similar shapes. In both cell lines CA exerted comparable effects within the whole tested pH range (pH 2–9.5). At low pH values, membranes of cells treated with CA showed an increase in positive *δ* values in comparison to control (untreated) ones, whereas at high pH values, cells treated with CA had less negative *δ* values compared to the untreated controls. The experimental curves reached a plateau in both acidic and basic pH. These alterations appeared to be time-dependent since more pronounced effects were observed for cells incubated for 48 h than 24 h. Moreover, post-hoc analysis revealed that the *δ* values of CA-treated and non-treated cells were statistically different (ANOVA, *p* ≤ 0.05; marks of statistical significance indicated in Table 1, but not on the Fig. 2 to preserve clarity of the data perception).

**Table 1:** Effect of caffeic acid on the acidic and basic functional groups concentrations and associations constants with H^+^ and OH^−^ ions of U118MG glioblastoma and DLD-1 colorectal adenocarcinoma cell membranes.

Cell line	Groups	Parameters			
		*C*_TA_^a^	*C*_TB_^b^	*K*_AH_^c^	*K*_BOH_^d^
		(10^−6^ mol/m^2^)	(10^−6^ mol/m^2^)	(m^3^/mol)	(10^7^ m^3^/mol)
U118MG	Control (24 h)	4.46 ± 0.05	1.22 ± 0.04	259.50 ± 1.22	3.27 ± 0.07
	+ 1 mmol/L CA	3.76 ± 0.06**	1.49 ± 0.06**	373.30 ± 1.14**	2.67 ± 0.09**
	Control (48 h)	4.74 ± 0.07	1.15 ± 0.07	271.50 ± 1.31	1.98 ± 0.04
	+ 1 mmol/L CA	3.51 ± 0.04**	1.37 ± 0.05	316.10 ± 1.44	1.79 ± 0.04
DLD-1	Control (24 h)	4.04 ± 0.06	1.05 ± 0.03	153.40 ± 1.32	0.89 ± 0.07
	+ 4 mmol/L CA	3.53 ± 0.05**	1.19 ± 0.06**	318.20 ± 1.24**	0.64 ± 0.05**
	Control (48 h)	4.32 ± 0.07	0.78 ± 0.06	319.00 ± 1.15	0.43 ± 0.01
	+ 4 mmol/L CA	3.06 ± 0.10**	1.01 ± 0.03**	382.40 ± 1.13**	0.39 ± 0.01**

^a^Concentration of functional acidic groups.

^b^Concentration of functional basic groups.

^c^Association constant with hydrogen ions.

^d^Association constant with hydroxyl ions. Statistical difference in comparison to control.

**p < 0.01.

Importantly, theoretical data concerning membrane surface charge densities seemed to fit the experimental ones pretty accurately within the whole pH range in both U118MG and DLD-1 cells. As such, based on the comparison of observed results, it can be concluded that the mathematical model quantitatively characterizing acid–base equilibria in natural cell membranes ("Electrophoretic light scattering measurements" Section) correctly describes interactions in analyzed systems.

### Effect of caffeic acid on the parameters characterizing U118MG and DLD-1 cell surfaces

Based on the model presented in "Electrophoretic light scattering measurements" Section, other parameters characterizing cell membrane surface can also be determined (Eqs. (5) and (6)). These are the total surface concentrations of functional acidic (*C*_TA_) and basic (*C*_TB_) groups as well as their average association constants of negatively charged groups with hydrogen ions (*K*_AH_) and positively charged groups with hydroxyl ions (*K*_BOH_). Table 1 presents the numeric values ± SD of the parameters determined for treated and untreated U118MG and DLD-1 cell lines. Of note, treatment of both cancer cell lines with CA caused significant changes in all calculated parameters compared to the untreated controls. Incubation with caffeic acid resulted in decreased values of *K*_BOH_ and increased values of *K*_AH_. Moreover, CA-stimulated cells were characterized by lower *C*_TA_ values and higher *C*_TB_ values. These results may suggest that CA-dependent changes in values of the analyzed electrochemical parameters of both U118MG and DLD-1 cells might occur due to the appearance or disappearance of certain functional groups on the surface of cell membranes.

### Effect of caffeic acid on the surface charge of phospholipid liposomal membranes

**Figure 3:**
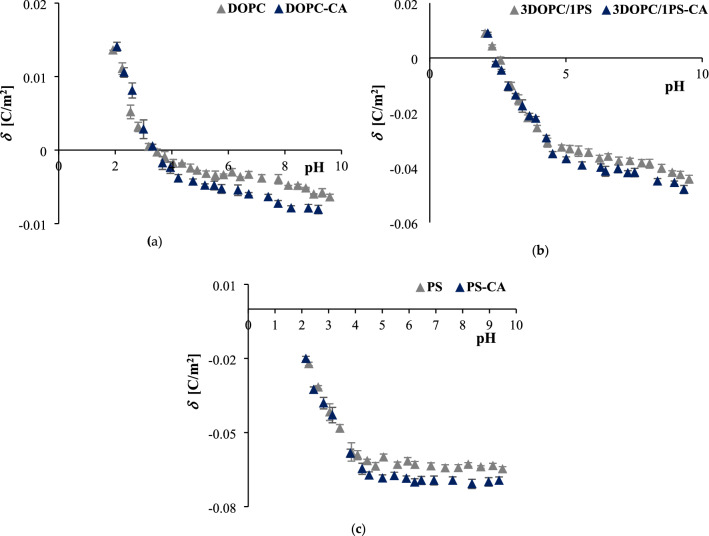
pH-dependent surface charge density curves of (**a**) DOPC, (**b**) 3DOPC/1PS, (**c**) PS liposomal membranes as a function of 0 (grey), and 1 mmol/L (navy blue) concentration of caffeic acid.

Physicochemical properties of liposomes, such as *δ* play a crucial role in the determination of their biological interactions. In this respect, the electrophoretic mobility of three different SUVs (pure zwitterionic DOPC, pure negatively charged PS, and binary mixtures of 3DOPC/1PS), as well as the liposomes containing CA (1 mmol/L), were determined by the ELS method. The measurements were performed in a function of hydrogen ion concentration. Based on the electrophoretic mobility values, surface charge densities were calculated from Eq. (1). Figure 3 depicts the surface charge densities of DOPC, 3DOPC/1PS, and PS liposomes formed from pure lipids and modified with CA as a pH function of *C*_NaCl_ = 155 mmol/L.

The dependencies presented in Fig. 3 are similar for all SUVs. Along with an increase in pH, the surface charge density of pure and CA-modified DOPC, 3DOPC/1PS, PS liposomal membranes becomes more and more negative. However, in the case of both unmodified and CA-modified PS liposomes the isoelectric point was not achieved and the curves reached a plateau at pH > 4 (Fig. 3c). The presence of CA in all liposomal systems led to the statistically significant increase in the negative surface charge density compared to pure membranes (Fig. 3a–c). These changes were the most visible for pH > 4 for DOPC membranes (Fig. 3a). In strongly acidic pH no statistically significant difference between the *δ* of the pure and CA-modified membranes were observed for all analyzed SUVs (ANOVA, *p* ≥ 0.05, marks of statistical significance indicated in Table 2, but not on the Fig. 3 to preserve clarity of the data perception).

**Table 2:** Physicochemical characterization of DOPC, 3DOPC/1PS, and PS SUVs, pure and modified with 1 mmol/L of caffeic acid, at pH 3.0 and pH 7.4.

pH	SUV composition	Diameter [nm]	Zeta potential [mv]	Polydispersity index (PDI)
3.0	DOPC	102.0 ± 5.4	1.18 ± 0.24	0.295
	DOPC-CA	113.2 ± 3.0**	1.67 ± 0.32**	0.177
	3DOPC/1PS	127.0 ± 3.3	− 6.85 ± 0.51	0.201
	3DOPC/1PS -CA	138.3 ± 3.7**	− 7.49 ± 0.45**	0.144
	PS	138.1 ± 4.1	− 26.74 ± 0.61	0.215
	PS-CA	149.7 ± 3.2**	− 27.74 ± 0.58**	0.143
7.4	DOPC	113.8 ± 2.0	− 2.54 ± 0.33	0.286
	DOPC-CA	125.9 ± 3.8**	− 4.22 ± 0.46**	0.294
	3DOPC/1PS	120.1 ± 3.0	− 25.34 ± 0.43	0.129
	3DOPC/1PS -CA	130.2 ± 3.5**	− 27.94 ± 0.60**	0.118
	PS	129.7 ± 3.1	− 42.92 ± 0.67	0.135
	PS-CA	140.1 ± 4.4**	− 46.38 ± 0.73**	0.233

Statistical difference in comparison to pure SUVs **p < 0.01.

### Effect of caffeic acid on the physicochemical parameters of phospholipid liposomal membranes

Evaluation of the size of nanoparticles as well as their zeta potential is crucial for the application of the nanosystems in biomedicine. Zeta potential is a physicochemical parameter that depends on the surface charge and is important for describing the stability of the nanoparticles in suspension. Moreover, both size and ζ of liposomes are important factors in the evaluation of their effectiveness as drug delivery systems and the prediction of their intracellular uptake. Thus, the size of the nanosystem may influence pharmacokinetics and tissue distribution of the drug. In this respect, we determined the set of physicochemical properties of SUVs including diameter, *ζ*, and PDI and collected them in Table 2. To determine the size and the PDI of the liposomes the DLS technique was employed, whereas for the evaluation of *ζ* the ELS technique was used. The size of the liposomes was depicted as the mean diameter. The values of the PDI typically range between 0.0, for fully monodispersed samples, to 1.0, for entirely polydispersed nanoparticles.

According to our results, the diameter of pure and CA-modified liposomes ranged between 102.0 (DOPC, pH = 3.0) and 149.7 nm (PS-CA, pH = 3.0), indicating that the obtained models can quite precisely mimic the structure and curvature of biological membranes. Moreover, moderate changes in size were noticed upon the addition of CA in either pH = 3.0 or physiological pH conditions. Interestingly, in acidic pH zwitterionic DOPC liposomes (both pure and modified with CA) tend to have smaller diameters than those exposed to physiological pH, whereas negatively charged DOPC/PS and PS liposomes showed larger diameters in pH = 3.0 compared to pH = 7.4. Polydispersity index values ranged from 0.118 (3DOPC/1PS-CA, pH = 7.4) to 0.295 (DOPC, pH = 3.0), revealing moderate size homogeneity of these vesicles.

The sign and magnitude of ζ are determined by the net charge accumulated on the liposome surface. According to the data presented in Table 2, *ζ* was strongly dependent on the lipid composition of the membrane and the pH of the electrolyte solution. In pH = 3.0 the values of *ζ* ranged from 1.18 mV for DOPC liposomes to − 27.74 mV for PS vesicles. Conversely, in physiological pH these values ranged from − 2.54 mV for DOPC to − 42.92 mV for PS liposomes. These results show that the effect of CA on zeta potential values was only noticeable in physiological pH. Thus, more negative *ζ* was found in DOPC, 3DOPC/1PS, and PS liposomes with the polyphenol presence. In pH = 3.0, this effect seemed to be insignificant.

### Effect of caffeic acid on the impedance parameters of bilayer lipid membranes

Electrochemical impedance spectroscopy is used in a plethora of biological^30–32^ and non-biological applications^33^. EIS is often applied as an analytic tool to support or confirm data obtained in other electrochemical methods such as cyclic or quadratic voltammetry. In our studies, we applied EIS to identify the changes in *C*_m_ and *R*_m_ values caused by the addition of CA into the lipid membrane solution. CA was applied at the concentration of 1 mmol/L. For BLMs preparation, two different phospholipids, i.e., DOPC and PS were used. Unfortunately, bilayers comprised purely of PS were not able to form membranes stable enough to withstand EIS experiments, thus only DOPC and 3DOPC/1PS bilayers were tested.

**Figure 4:**
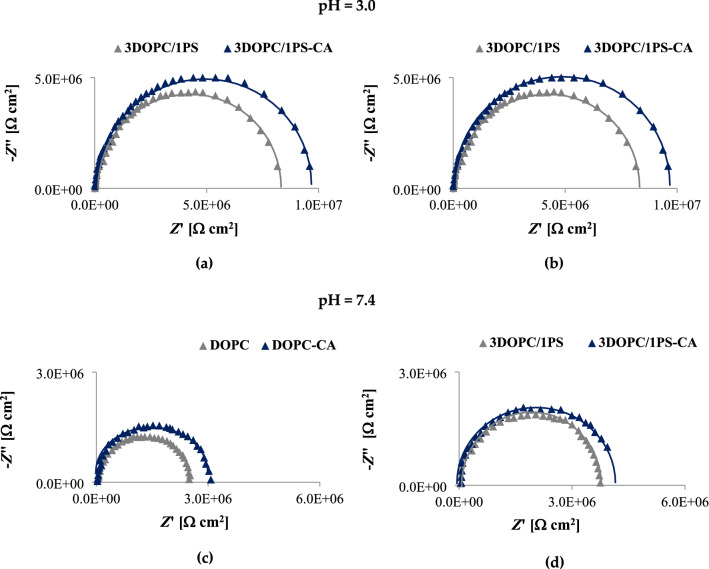
Impedance plots (experimental-dotted curve, and fitted-solid curve) obtained at pH = 3.0 (**a**,**b**) and pH = 7.4 (**c**,**d**) for: DOPC (**a**,**c**), and 3DOPC/1PS (**b**,**d**) bilayers as a function of 0 (grey) and 1 mmol/L (navy blue) concentration of caffeic acid.

The layout of the EIS shown in Fig. 4 demonstrates that the bilayer modification was successful since there is an increase in the Nyquist plot capacitive arc diameter after CA addition. Moreover, the statistically significant difference between the impedance parameters of pure and CA-modified BLMs started from the frequency of 25 Hz. The augmentation of the semicircle diameter in the Nyquist plot could be attributed to the steric and resistive effect of the phenolic acid attached to the membrane surface, corroborating the pattern observed in the ELS measurements.

Fitting procedure was performed to extract the impedance parameters values from the spectra and to validate the EIS data. The comparison of the theoretical and experimental impedance values in the frequency domain was done to investigate the influence of external factors on the EIS data. Figure 4 compares both sets of data. The overlapping of the plots indicates that the experimental impedance data agree with the theoretical values, and the alignment of the theoretical and experimental results indicates that the electrochemical response is causal, linear, and stable.

To mimic natural membranes and make a good modeling system, artificial bilayers should be defined by specific values of capacitance and resistance characteristic for natural cell membranes. The capacitance of the BLM can usually be considered as a parallel plate capacitor with *C* = ɛɛ_0_/*d*, where ɛ is the relative permittivity, ɛ_0_ is the permittivity of free space, and *d* is the membrane thickness. Taking a typical hydrophobic thickness of about 4 nm and a relative permittivity of 2–4, the membrane capacitance is expected to be 0.5–1 μF/cm^2^. Typical values of membrane resistance fit within the range of 10^4^–10^7^ Ω cm^2^. The values of both parameters fitting within these ranges have already been reported in many previous works^34–36^. Table 3 provides the values of the fitting parameters for the impedance spectra presented in Fig. 4. Noticeably, the *C*_m_ and *R*_m_ values obtained in our experiment were consistent with the values reported in the literature for other model bilayers. On the other hand, the Re values received from the measurements performed in both pH = 3.0 and pH = 7.4 were similar and mirror the results reported previously^13^.

**Table 3:** The fitting parameters of the equivalent circuit for the impedance plots.

pH	BLM composition	*R*_e_ [10^3^ Ω cm^2^]	*R*_m_ [10^6^ Ω cm^2^]	*C*_m_ [10^−6^ μF/cm]
3.0	DOPC	5.150 ± 0.402	6.535 ± 0.07	0.634 ± 0.02
	DOPC-CA	5.230 ± 0.370	7.222 ± 0.07**	0.644 ± 0.03
	3DOPC/1PS	5.191 ± 0.402	8.341 ± 0.08	0.652 ± 0.03
	3DOPC/1PS -CA	5.210 ± 0.411	9.562 ± 0.08**	0.661 ± 0.04
7.4	DOPC	5.172 ± 0.392	2.493 ± 0.08	0.627 ± 0.04
	DOPC-CA	5.198 ± 0.417	3.067 ± 0.06**	0.664 ± 0.02**
	3DOPC/1PS	5.183 ± 0.382	3.748 ± 0.05	0.643 ± 0.04
	3DOPC/1PS-CA	5.197 ± 0.409	4.073 ± 0.07**	0.671 ± 0.03*

Statistical difference in comparison to pure BLMs *p < 0.05, **p < 0.01.

Based on the data gathered in Table 3 and the values plotted in Fig. 4, it may be noted that CA modulates values of the impedance parameters of the BLMs at both pH = 3.0 as well as pH = 7.4. This allows us to conclude that this phenolic acid interacts with both neutral and anionic membranes at both acidic and physiological pH.

## Discussion

Given the importance of physicochemical properties of potential drug candidates, we first verified if CA fulfills the requirements of three different rules describing drug-like potential of chemical substances. These rules are Lipinski’s rule, Veber’s rule, and Ghose filter. According to Lipinski’s “rule of five”, a poor permeation or absorption of a drug is more probable when its molecule contains more than 5 H-bond donors, over 10 H-bond acceptors, the molecular weight (MW) greater than 500, and the calculated log P (*c*log *P*) greater than 5^37^. Subsequently, Veber’s rule for good bioavailability is determined by the polar surface area (TPSA), indicating TPSA ≤ 140 Å and ≤ 10 of rotatable bonds^38^. In consequence, Ghose et al. proposed the following characterization of a drug-like molecule: (1) an organic compound having *c*log *P* between − 0.4 and 5.6, a molar refractivity between 40 and 130, a MW of 160 g/mol to 480 g/mol; and atom count between 20 and 70; (2) possessing a combination of certain chemical groups such as benzene ring, heterocyclic ring, aliphatic amine, carboxamide group, alcoholic hydroxyl group, carboxy ester, or a keto group; (3) maintaining chemical stability in the physiological buffer^39^. CA fulfills all three aforementioned rules with MW = 180.04, *c*log *P* = 0.203, number of H-bond acceptors = 4, number of H-bond donors = 3, number of rotatable bonds = 2, TPSA = 77.76 Å, a molar refractivity = 50.41 and atom count = 21^40,41^. Thus, CA exhibits beneficial drug-like properties, which encouraged us to use this compound in pharmacooncology as a promising cytostatic agent of natural origin. Hence, we evaluated the cytotoxicity of CA in U118 MG and DLD-1 cells (Fig. 1), confirming good cytostatic potential in the analyzed cell lines, especially in higher CA concentrations.

Another important feature of drug candidates is their membrane permeability. Assuming the surface-bound location of CA in cell membranes, we used ELS technique to determine both experimental and theoretical values of pH-dependent surface charge density of membranes of U118 MG and DLD-1 cells (Fig. 2). CA-treated cells showed an increase in positive *δ* values at low pH and a decrease in negative *δ* values at high pH in comparison to control cells. In principle, unlike normal cells, cancer cells have a deregulated pH profile because their extracellular environment is typically acidic, while their intracellular space is alkaline^42^. Thus, the decrease in the negative charge of the analyzed biomembranes suggests that in cells incubated with CA, the acidic properties of the surface were reduced. Consequently, a shift in the isoelectric point of the cell membrane towards higher pH values was observed. In the native state, the cell surface is composed of charged proteins, glycocalyx carbohydrates, and lipids^43^. The decrease in the negative charge of the analyzed biomembranes suggests that in cells incubated with CA, the acidic properties of the surface were reduced. Such reduction could occur due to several reasons: (I) a decrease in the free fatty acids content, (II) an increase in the integral proteins level, (III) a translocation of anionic phospholipids, especially PS, to the inner part of the cell membrane, or (IV) a reduction in the sialic acid content, which is considered a marker of tumorigenesis. These alterations lead to a change in the number of functional groups exposed to the surface of the cell membrane, thus modulating its electrical properties. Overall, changes in values of the constants presented here: a decrease in the *C*_TA_ and *K*_BOH_ values, as well as an increase in the *C*_TB_ and *K*_AH_ values, are in agreement with other studies showing the same modulatory tendency in these physicochemical parameters of cancer cell surface upon exposition to chemotherapeutic agents^14,15^.

Heller et al.^43^ demonstrated that the charge density of the cell membranes of different cell lines after the removal of cell surface proteins by trypsinization was almost identical in various cell types. This is likely due to the consistent lipid bilayer properties typical for the majority of mammalian cells. Analysis of the curves presented in Fig. 2, indicates that DLD-1 cells showed lower positive *δ* values at low pH and diminished negative *δ* values at high pH in comparison to U118MG cells. This might be the result of the different content of cell surface proteins in both lines. These observations suggest that changes in surface charge of membranes of living cells indicate the differential composition of cell membranes in various cell types.

In pharmaceutical sciences, liposomes have been used to investigate the relationship between the biological activity of compounds and their interaction with lipid membranes. Therefore, we evaluated the affinity of CA to membranes using liposomes composed of DOPC, PS or a mixture of two of these phospholipids. DOPC is an unsaturated glycerophospholipid that is often used for liposomal encapsulation of chemotherapeutic agents^44^. PS is the main class of acidic phospholipids, which constitutes 13–15% of phospholipids in the human cerebral cortex^45^. The results of the microelectrophoretic measurements shown in Fig. 3 clearly indicate that CA does not affect the *δ* of liposomal membranes at acidic pH. Supposedly, at acidic pH this compound is able to dissolve in the membrane and permeate it. However, in neutral and alkaline pH, a carboxylic acid group of CA can potentially anchor it to the polar heads of membrane phospholipids confining its action to the more external region of the membrane. As a result, CA cannot disrupt the lipid structure, but instead, it causes significant changes in the electrical charge values accumulated on the surface of the membrane. Regarding the CA's p*K*_COOH_ value of 4.7 ± 0.1^46^, it is reasonable to speculate that at neutral and alkaline pH the carboxylic group is completely ionized and the negative charge of CA molecule is presumably oriented to the positive pole of zwitterionic DOPC headgroup. This hypothesis is supported by our results, showing increasingly negative values of the surface charge of the membranes along with the addition of the CA. A similar trend has also been reported for other negatively charged compounds^47^.

Additionally, the physicochemical properties of the prepared liposomes such as diameter, PDI, and zeta potential, were determined at two different pHs (3.0 and 7.4) and are presented in Table 2. As expected, the addition of CA resulted in a slight increase in the average size of the liposomes. A similar trend was previously observed for liposomes constructed with the same phospholipids with different hydroxycinnamic acids^13^. Interestingly, other reports have shown increased, unaffected, or even decreased particle sizes after the incorporation of different bioactive compounds into liposomes^48^ and references therein]. Thus, it can be concluded that the introduction of certain compounds into the liposome does not have to significantly affect the particle sizes. Furthermore, the PDI of the particle size distributions reported in the aforementioned studies ranged from 0.05 to 0.31. Hence, the PDI obtained in our study fits well within the range of values reported in previous works.

Liposomes offer a great potential in CA entrapment, providing unchanged polyphenol bioactivity. According to Caddeo et al.^49^, in general, liposomal encapsulation is not significantly influenced by the lipids used for the preparation of the liposomes. Dejeu et al.^50^ determining of entrapment efficiency showed that, regardless of the composition of phospholipids (PC, DMPC and DPPC), the percentage of CA entrapment was up to 76%. Moreover, it was observed that the release of entrapped CA occurs gradually. In the first eight hours the release is at its high (over 80%), after which it is getting reduced^50^. Katuwavila et al.^51^ prepared caffeic acid-loaded egg phosphatidylcholine liposomes with a similar encapsulation efficiency of nearly 70%. Such high encapsulation efficiency was believed to be related to the poor water solubility of caffeic acid, and thus its complete incorporation into the lipid bilayer. These liposomes were able to achieve a cumulative release of 71% of entrapped CA within 7 h in PBS solution (pH 7.4), whereas 95% of free caffeic acid diffused within 4 h. Morphological analysis of these liposomes by SEM showed spherical shaped 3D globular vesicles. Zaremba-Czogalla et al*.*^52^ encapsulated caffeic acid derivative into liposomes composed of soybean phosphatidylcholine and DSPE-PEG2000 obtaining 93% of the entrapment efficiency. The loaded liposomes had larger size compared to unloaded ones. Moreover, said liposomes appeared to be slightly smaller in size in the TEM images than the sizes determined from DLS measurements. This is due to the fact that DLS measurements are performed for liposomes in solution, while the TEM technique is used to estimate the size of dried vesicles, which are smaller than in the hydrated state.

As shown in the literature, pH affects the release, solubility and permeation of acidic drugs^53,54^. Although the interactions of these compounds with membranes at physiological pH have been well-characterized, the molecular mechanisms underlying the above interactions at acidic pH are still a matter of study. Additionally, it was reported that at slightly acidic pH (4.0 or 5.5), the release of phenolic acids is higher as compared to the release tested at neutral pH (i.e., 7.4)^53,54^. Lúcio et al*.*^55^ utilized synchrotron small-angle X-ray scattering (SAXS) and wide-angle X-ray scattering (WAXS) to access the structural modifications arising from the interaction of weak acids (tolmetin and indomethacin) and DPPC membranes, at acidic conditions. At pH 5, those compounds gain negative charge and, when used in low doses, may partially penetrate within the polar region of the phospholipids, hence enhancing the membrane disorganization. This is consistent with the results of WAXS measurements, in which a dislocation of both Bragg peak positions as well as a diminished correlation length can be seen. However, for higher concentrations of tolmetin and indomethacin, the WAXS profiles show the existence of a sharp reflection typical for the hexagonal packing of nontilted chains. This packing, by reducing the area requirement of the headgroups, would lead to an increase of the bilayer thickness. Conversely, SAXS measurements indicate a reduction of the bilayer thickness through the penetration of the drug into the headgroup region, thereby increasing the area requirement of the headgroups and leading to an interdigitation of the acyl chains to decrease the mismatch of area requirements between heads and tails. Such an interdigitation of the acyl chains is energetically more favorable than a strong tilting of the chains.

The capacitance and resistance measurements performed by EIS technique on DOPC and 3DOPC/1PS BLMs (Fig. 4, Table 3), revealed that the incorporation of CA into the bilayers was pH-dependent. At pH = 3.0, the CA insertion caused an increase in the membrane resistance, and hence reduced conductivity. An increase in the *R*_m_ value suggests that this phenolic acid enhances the ordering and reduces the dynamics of the alkyl chains of the phospholipids in membranes. Unlike changes in the *R*_m_ values, changes in the *C*_m_ were less evident in Nyquist plots so that no significant differences were apparent between successive EIS measurements. Analysis of the *C*_m_ values determined from the equivalent circuit model overlapping with Nyquist indications reveals a slight increase in membrane capacity in the presence of phenolic acid, which can be associated with a decrease in membrane thickness. This in turn, together with an increase in the resistance, may be indicative of an ordering and stabilizing effect of CA on the alkyl chains of membrane-building phospholipids occurring when the environment surrounding the membrane is characterized by the acidic pH.

In contrast, at pH equal to 7.4, the membrane resistance was almost not affected, since CA did not perturb the molecular packing of the hydrocarbonic acyl chains of the phospholipids. Only a slight increase in the *R*_m_ value may thus indicate that at physiological pH this phenolic acid is incapable of permeating the bilayer structure efficiently. At the same pH, an increase in membrane capacity of CA-simulated membranes, compared to the values obtained at acidic pH, was much more pronounced. These significant changes in *C*_m_ suggest the adsorption of CA on the BLMs surface. Analysis of the impedance spectra allows to imply that the adsorption of CA on the membrane did not result in any additional time constant, which was also stated in the case of protein adsorption^56^.

Furthermore, the comparison of the effect of the polar lipid headgroup and the impedance parameters of phenolic acid-modified BLMs, suggests that CA interacts with the negatively charged lipid headgroup of PS to a lower extent than with DOPC membranes. This finding is not surprising considering that at physiological pH CA has a negative charge that may generate charge repulsion between the two carboxylate functions of the serine in PS. In contrast, in acidic pH, where CA is neutral and completely liposoluble, differences in the interaction between CA and DOPC or PS cannot be clearly identified.

Modern biomedical and pharmacological research concentrate on involving multidirectional analyses for maximal improvement of the therapeutic outcomes. Thus, as an attempt to cover various aspects of membrane-CA interactions, the microelectrophoretic mobility measurements and electrochemical impedance spectroscopy were applied to study the effects of CA on the electrical properties of cancer cells and biomimetic membranes. Our results demonstrated that CA caused significant changes in the values of electrical charge accumulated on the surface of cellular membranes within the whole range of the analyzed pH. In contrast, the change in surface charge of liposomal membranes after CA treatment was detected only in alkaline pH solutions, whereas the of liposomal membranes at acidic pH was not affected by CA. This allows us to conclude that at acidic pH this compound is able to dissolve in the liposomal membrane and penetrate it. However, in neutral and alkaline pH, CA can anchor to the membrane surface. The impedance data showed an increase in values of both the electrical capacitance and the electrical resistance, indicating that CA can be partially inserted into the phospholipid bilayers.

Taking into account certain limitations of our study, further investigations of the physicochemical parameters of membranes and their influence on the therapeutic efficiency of potential drug candidates are highly required. Since the electrical parameters of living cells are the result of the mutual interactions between their components, the existence of various proteins should be taken into account when considering the influence of CA on the electrical properties of cell membranes. Of note, the cell surface proteins are usually less anionic than the phospholipid bilayer, thus the charge generated by these proteins might mediate the overall cell surface charge^43^.

Altogether, although data on physicochemical properties of potential drugs is constantly being collected, a hefty amount of information is still lacking. In his respect, further studies are necessary before the application of physicochemical analyses becomes a standard procedure in pharmacology pharmacotherapy.

## Conclusions

In the present paper, we investigated the pH-dependent incorporation of caffeic acid into liposomes, bilayer lipid membranes, and human glioblastoma and human colorectal adenocarcinoma cell membranes. Our results demonstrate that CA changes electrochemical parameters such as surface charge density, electrical capacitance, or electrical resistance of almost all analyzed membranes via localization in the polar head group region at physiological and alkaline pH or intercalation between the flexible acyl chains of the phospholipids in acidic pH. Using the microelectrophoresis and electrochemical impedance spectroscopy techniques, we have shown that modulation of certain experimental conditions (e.g. lowering the pH) may bring valuable knowledge about the tested compound. As such, the determination of physicochemical properties of bioactive compounds can give insight into their membrane-permeation abilities. Altogether, our results suggest that CA presents favoring drug-like properties, fulfilling physicochemical requirements for being orally bioavailable and membrane-permeable polyphenol. Simultaneously, reduced viability confirms that CA enters the cells and results in perturbed proliferation of U118MG and DLD-1 cancer cell lines. Our research is one of the first reports trying to coordinate cell-based and physicochemical studies in order to unravel the complicated nature of membrane-related functioning of CA.

## Abbreviations

CA: Caffeic acid
DOPC: 1,2-Dioleoyl-sn-glycero-3-phosphocholine
PC: Phosphatidylcholine
PS: Phosphatidylserine
SUV: Small unilamellar vesicle
BLM: Bilayer lipid membranes
EIS: Electrochemical impedance spectroscopy
*C*_m_: Membrane capacitance
*R*_m_: Membrane resistance
DLS: Dynamic light scattering
ELS: Electrophoretic light scattering
*δ*: Surface charge density
*C*_TA_: Concentration of functional acidic groups
*C*_TB_: Concentration of functional basic groups
*K*_AH_: Association constant with hydrogen ions
*K*_BOH_: Association constant with hydroxyl ions
PDI: Polydispersity index
*ζ*: Zeta potential
MW: Molecular weight
*c*log *P*: Calculated n-octanol/water partition coefficient
TPSA: Polar surface area
